# Anti–Inflammatory and Antioxidant Properties of the Ethanol Extract of *Clerodendrum Cyrtophyllum* Turcz in Copper Sulfate-Induced Inflammation in Zebrafish

**DOI:** 10.3390/antiox9030192

**Published:** 2020-02-25

**Authors:** Thu Hang Nguyen, Hong Diep Le, Thanh Nguyen Thi Kim, Hai Pham The, Thi Mai Nguyen, Valérie Cornet, Jérôme Lambert, Patrick Kestemont

**Affiliations:** 1 Research Unit in Environmental and Evolutionary Biology (URBE), Institute of Life, Earth and Environment (ILEE), University of Namur, 5000 Namur, Belgium; nguyenthu@student.unamur.be (T.H.N.); ntmai.ntts@vnua.edu.vn (T.M.N.);; 2 Pharmacology Department, Hanoi University of Pharmacy, Hanoi 100000, Vietnam; 3 VNU University of Science, Vietnam National University, Hanoi 100000, Vietnam; dieplh@hus.edu.vn (H.D.L.); kimthanh_bio@vnu.edu.vn (T.N.T.K.); phamthehai@vnu.edu.vn (H.P.T.); 4 Faculty of Fisheries, Vietnam National University of Agriculture, Hanoi 100000, Vietnam

**Keywords:** Anti-inflammation, oxidative stress, antioxidant, CuSO_4_, zebrafish larvae, *Clerodendrum cyrtophyllum*

## Abstract

Oxidative stress and inflammation are commonly present in many chronic diseases. These responses are closely related to pathophysiological processes. The inflammatory process can induce oxidative stress and vice versa through the activation of multiple pathways. Therefore, agents with antioxidant and/or anti-inflammatory activities are very useful in the treatment of many pathologies. *Clerodendrum cyrthophyllum* Turcz, a plant belonging to the Verbenaceae family, is used in Vietnamese traditional medicine for treating migraine, hypertension, inflammation of the throat, and rheumatic arthritis. Despite its usefulness, studies on its biological properties are still scarce. In this study, ethanol extract (EE) of leaves of *C. cyrtophyllum* showed protective activity against CuSO_4_ toxicity. The protective activity was proven to relate to antioxidant and anti-inflammatory properties. EE exhibited relatively high antioxidant activity (IC_50_ of 16.45 µg/mL) as measured by DPPH assay. In an in vivo anti-antioxidant test, three days post fertilization (dpf) zebrafish larvae were treated with different concentrations of EE for 1 h and then exposed to 10 µM CuSO_4_ for 20 min to induce oxidative stress. Fluorescent probes were used to detect and quantify oxidative stress by measuring the fluorescent intensity (FI) in larvae. FI significantly decreased in the presence of EE at 5 and 20 µg/mL, demonstrating EE’s profound antioxidant effects, reducing or preventing oxidative stress from CuSO_4_. Moreover, the co-administration of EE also protected zebrafish larvae against oxidative damage from CuSO_4_ through down-regulation of *hsp70* and *gadd45bb* expression and upregulation of *sod*. Due to copper accumulation in zebrafish tissues, the damage and oxidative stress were exacerbated overtime, resulting in the upregulation of genes related to inflammatory processes such as *cox-2*, *pla2*, *c3a*, *mpo*, and pro- and anti-inflammatory cytokines (*il-1ß*, *il-8*, *tnf-α*, and *il-10*, respectively). However, the association of CuSO_4_ with EE significantly decreased the expression of *cox-2*, *pla2*, *c3a*, *mpo*, *il-8*, and *il-1ß*. Taken together, the results suggest that EE has potent antioxidant and anti-inflammatory activities and may be useful in the treatment of various inflammatory diseases.

## 1. Introduction

Oxidative stress and inflammation contribute to the pathogenesis of many chronic diseases such as diabetes, cardiovascular diseases, neurodegenerative diseases, cancer, and aging [1]. They are tightly linked with one another. If inflammation is the primary event, the activated macrophages and neutrophils liberate large amounts of reactive species at the site of inflammation [2]. Consequently, oxidative stress will develop and further increase inflammation. Conversely, if oxidative stress appears, the overproduced reactive oxygen/nitrogen species can induce inflammation through upregulation of pro-inflammatory gene expression [1,3]. Inflammation will eventually further accentuate oxidative stress. In clinical practice, identification and treatment of the primary abnormality are very important. However, the relationship between inflammation and oxidative stress is interdependent; treating only the primary abnormality may not always be successful [1]. Therefore, finding agents that inhibit both ROS production and inflammatory mediators has been considered a promising strategy for preventing or treating diseases associated with chronic inflammatory conditions.

Zebrafish (*Danio rerio*) have been widely used in developmental biology and drug discovery. Several characteristics make zebrafish a convincing tool in drug discovery, such as low maintenance costs, rapid embryogenesis, transparency, high similarity between the human and zebrafish genomes, etc. [4]. More specifically related to this research, oxidative and inflammatory responses can be robustly and easily induced and visualized in zebrafish, particularly in the early stages [5]. Copper is an essential micronutrient but misregulation of intracellular levels can become toxic to many cell types. Under inflammatory conditions, serum copper levels increase and trigger oxidative stress responses that activate inflammatory responses [5]. The zebrafish copper-induced inflammation model has been used previously and presents several advantages as it is a non-invasive and sterile method, in comparison to methods involving physical damage and the use of infectious agents, besides its gentle manipulation of the larvae [6]. In this study, we used CuSO_4_ as an agent to induce an inflammatory response through the oxidative stress reaction to study the antioxidant and anti-inflammatory effects of ethanol extracts of *Clerodendrum cyrthophyllum* Turcz.

*C. cyrthophyllum*, a plant belonging to the Verbenaceae family, is widely distributed in tropical countries such as Vietnam, China, India, Japan, Korea, and Thailand [7]. In Vietnam, it is used for treating migraine, hypertension, inflammation of the throat, and rheumatic arthritis. In several studies, phenolic acids and flavonoids have been isolated from various plant parts [8]. Total phenols and flavonoids displayed good antioxidant activity and potential anti-inflammatory activity in several experimental models [9]. Investigations on this plant suggest that it has potential as an alternative treatment for inflammatory diseases. However, further investigation is needed to identify the exact mechanism of action and pathways that are modulated by this plant. In this study, we investigated the antioxidant and anti-inflammatory effects of the ethanol extract of *C. cyrthophyllum* using the zebrafish copper-induced inflammation model to elucidate the molecular mechanisms to explain its effects, and provide scientific evidence for using it in traditional medicine.

## 2. Materials and Methods 

### 2.1. Chemicals

2,2-Diphenyl-1-picrylhydrazyl (DPPH), quercetin, 2′,7′-dichlorodihydrofluorescein diacetate (H2DCFDA), copper sulfate pentahydrate (CuSO_4_·5H_2_O), and pyrocatechol violet (PV) were purchased from Sigma Chemical Co. (St. Louis, MO, USA). All other reagents were of the highest commercially available grade.

### 2.2. Preparation of the Ethanol Extract of Clerodendrum cyrtophyllum Turcz (EE)

#### 2.2.1. Plant Collection

Leaves of Clerodendrum cyrtophyllum Turcz Verbenaceae were collected from northern provinces of Vietnam in June 2018, by Nguyen Kim Thanh from Vietnam National University (VNU-BIOL). A voucher specimen (HNU 024106) was deposited at the Botanical Museum of Hanoi, University of Science. Leaves were cleaned and dried for 72 h to a constant weight using a hot air oven at 40 °C before extraction. Dried leaves were then ground in a blender to fine powder (size smaller than 0.5 mm). 

#### 2.2.2. Preparation of Total Extract

Given that polar (aqueous) extracts are used in traditional practice, we selected ethanol to extract polar secondary metabolites, minimizing the extraction of mineral salts and polysaccharides. For this, 100 g of dried leaf powder was extracted with 1000 mL 95% ethanol at 60 °C in a water bath with 360 rpm agitation for 4 h. The extract was then filtered and collected. The residue was extracted another two times using the same procedure. The collected ethanol extracts were then combined, concentrated on a rotary evaporator (40 °C) under reduced pressure, and lyophilized to obtain the crude extract. The yield of extract was 11.3% relative to the dried leaf powder. The crude extract was stored at −20 °C until use. After the extraction, the EE was analyzed with various analytical techniques including colorimetric methods, HPTLC (high performance thin layer chromatography), and mass spectrometry analyses. The extract presents a concentration of total phenolic compounds of 23.3 ± 1.5 GAE mg/g and flavonoid of 2.97 ± 0.01 QE mg/g expressed in dry weight of leaves material.

### 2.3. Fish and Experimental Conditions

The adult wild–type AB zebrafish (*Danio rerio*) were maintained in a recirculating ZebTec housing system (Techniplast) at 28 °C with a 12:12 h (light:dark) photoperiod. Conductivity was maintained at approximately 500 μS/cm, pH 7.2. Fish were fed ad libitum three times daily. The day before mating, males and females (2:2) were placed in spawning tanks. Naturally spawned embryos were obtained within 30 min after the lights were switched on in the morning. The embryos were selected visually using a binocular microscope and only fertilized and normally developed eggs were selected. Selected eggs were placed in embryo medium at 28 °C. Embryo medium was renewed every 24 h. Hatched larvae at three days post fertilization (dpf) were used for subsequent experiments. Since zebrafish larvae below 120 h old are not considered animals [10], no animal test authorization is required according to European legislation (EU Directive, 2010/63/EU). All experiments were terminated at 120 h post fertilization (hpf). The term “larvae” refers to hatched embryos up to 120 hpf that are using up the yolk-sac reserves and do not yet feed externally (also called yolk-sac larva, early larva, pre-larva, or eleuthero embryos) [10].

### 2.4. Experimental Design

#### 2.4.1. Preparation of Test Samples

The dry ethanol extract of *Clerodendrum cyrtophyllum* Turcz ***(EE)*** was dissolved in DMSO (dimethyl sulfoxide) to make a stock solution of 50 mg/mL. The stock solution was diluted to different concentrations using fresh larvae medium (dechlorinated water with conductivity approximately 500 μS/cm, pH 7.2) so that the final concentration in each experimental well was 5, 20, and 40 µg/mL. 

#### 2.4.2. Protective Effect of EE against CuSO_4_ Toxicity in Zebrafish Larvae

Mortality was used as an initial endpoint to characterize and evaluate the protective effect of the EE against CuSO_4_ toxicity in zebrafish larvae. Zebrafish larvae 3 dpf at a density of twenty larvae per well were incubated with EE at different doses (5, 20, and 40 µg/mL) for 1 h and then stimulated with a lethal dose (20 µM) of CuSO_4_ for 24 h. All treatments were performed in six-well culture plates. The parameter assessed was mortality rate at 24 h. 

#### 2.4.3. Anti-Oxidant Effect of EE

##### In Vitro Antioxidant Tests: Measurement of DPPH Radical Scavenging Capacity 

The ability of the prepared extract to scavenge DPPH radicals was determined by the method described by Cheng-zhong et al. (2013) [11] with slight modifications. Briefly, a 20 µL solution of EE in different concentrations (5, 10, 25, 50, 100, 250, and 500 µg/mL) was added to 180 µL methanol solution of DPPH (0.1 mM). Quercetin was used as a positive control and methanol was used as a blank. All reaction mixtures were shaken and incubated in the dark for 30 min at room temperature. The absorbance was then measured at 517 nm using a UV-Vis spectrophotometer. All samples were analyzed in triplicate. The DPPH scavenging ability of the plant extracts was calculated using the following equation:

%Scavenging Activity = (Abs control − Abs sample) × 100/(Abs control)

where Abs control is the absorbance of the blank and Abs sample is the absorbance of the sample.

The antioxidant activity was reported in terms of IC_50_ (concentration of extract necessary to decrease the initial concentration of DPPH by 50%).

##### Cu^2+^ Chelation Ability

The ability of EE to chelate Cu^2+^ was assessed using the method described by Santos et al. (2017) that employs pyrocatechol violet (PV) as the chromogen agent [12]. Briefly, in each well, 30 µL solution of EE in different concentrations (5, 10, 25, 50, 100, 250, and 500 µg/mL) or water (control) were mixed with 200 µL of sodium acetate buffer (50 mmol/L, pH 6.0). Then, 30 µL of a 100 mg/L CuSO_4_.5H_2_O solution were added in each well and let react for 2 min. After 2 min, 8.5 µL of solution pyrocatechol violet 2 mmol/L were added to initiate the reaction. All reaction mixtures were shaken and incubated for 10 min at 25 °C. The absorbance was then measured at 632 nm using a UV-Vis spectrophotometer. Cu^2+^ chelating ability of EE was calculated as:

Cu^2+^ chelating ability of EE (%) = [(Abs control − Abs sample)/Abs control] × 100.

##### In Vivo Antioxidant Test

Generation of reactive oxygen species was determined using the membrane-permeable fluorescent dye 2′,7′-dichlorodihydrofluorescein diacetate (H2DCFDA). H2DCFDA is hydrolyzed inside cells to the non-fluorescent compound 2′,7′-dichlorodihydrofluorescein, which emits fluorescence when oxidized to 2′,7′-dichlorofluorescein (DCF). Thus, the fluorescence emitted by DCF directly reflects the overall oxidative status of a cell [13,14]. Zebrafish larvae (3 dpf) were transferred into 12-well plates containing 940 µL embryo media, 6 larvae per well. Groups of 6 hatched larvae were treated with EE at different doses of 5, 20, and 40 µg/mL or 100 mM quercetin for 1 h and then exposed to 10 µM CuSO_4_ for 20 min to induce oxidative stress. After 20 min of exposure, larvae were washed twice with fresh larvae medium and incubated with H2DCFDA (2′,7′-dichlorodihydrofluorescein diacetate) solution (20 µg/mL) for 1 h in the dark at 28 ± 1 °C. After incubation, zebrafish larvae were washed 3 times in fresh larvae medium, anesthetized with 0.0003% MS-222 (tricaine methane-sulfonate), and transferred to flat 24-well plates (one larva per well) before observation and photography under a fluorescence microscope. Each larva was placed on the lateral side with the head preferably on the left side with respect to the observer. Fluorescence intensity of an individual larva was quantified using Autovision software and a BD pathway 855 system (BD Biosciences). Larvae exposed to CuSO_4_ but not treated with EE were used as controls.

The stock solutions of H2DCFDA were prepared in DMSO. The solutions were protected from light and stored at −20 °C until used for the analysis. Working solutions were prepared on the day of the experiment.

##### Quantitative Real–Time PCR

Three days post fertilization zebrafish larvae were transferred into 12-well plates containing 940 µL embryo media, 20 larvae per well. A group of 20 hatched larvae was first treated with EE at different doses of 5, 20, and 40 µg/mL for 1 h and then exposed to 10 µM CuSO_4_ (a concentration capable of inducing oxidative stress and inflammation) for 4 h. After 4 h, larvae were collected and stored at –80 °C for qPCR analysis to evaluate the effects of EE on the expression of genes related to oxidative stress, such as *sod*, *gpx4*, *hsp70*, and *gadd45bb*.

##### Total RNA Extraction, DNase Treatment and Reverse Transcription

Total RNA was extracted from these larvae using Trizol Reagent solution (Ambion, Thermo Fisher Scientific, Massachusetts, United states) following the manufacturer’s instructions. The pellet containing RNA was dried and resuspended in 100 µL of RNase-free water. The concentration of total RNA was determined spectrophotometrically at A_260_/A_280_ and A_260_/A_230_ nm using a NanoDrop^TM^ 1000 (Thermo Scientific). RNA integrity was checked by denaturating gel electrophoresis (1.2% agarose). The extracted RNA samples were then subjected to DNase treatment (DNA-*free*™ DNA Removal Kit, Invitrogen) to avoid DNA contamination. One microgram of total RNA was reverse-transcribed into double-stranded cDNA using the RevertAid RT kit (Thermo Scientific) according to the manufacturer’s instructions.

The resulting cDNA was used to measure the expression of 5 oxidant genes in a real-time quantitative polymerase chain reaction (qPCR). Two housekeeping genes (β-actin and elongation factor 1α (*efl1-α*)) were used as references. The expression of genes encoding *sod*, *cat*, *gpx4*, *hsp70*, and *gadd45bb* was evaluated. The list of specific primers used is given in Table 1. Primers were designed on Primer3 software. Amplifix software was used to check the quality of the primers. The efficiency of each primer was confirmed by RT-PCR.

RT-PCR was performed using an ABI StepOnePlus Real-Time PCR system (Applied Biosystems). Five microliters of 25-fold diluted cDNA template was mixed with 5 μL of each primer (5 μM) and 10 μL of SYBR green PCR master mix (Applied Biosystems) to a final volume of 20 μL. The standard cycling conditions were 95 °C for 10 min, followed by 40 cycles of 95 °C for 30 s and 60 °C for 30 s. All reactions were performed as technical triplicates. For analysis, a standard curve of a pool of the cDNA of all samples was constructed following the absolute quantification method (Bio-Rad) [15]. The equation for the linear regression line

CT = m(log quantity) + b.

where b is the y-intercept and m is the slope of the linear regression. Based on the equation for the linear regression, we can determine the quantity of an unknown sample:

$$\mathrm{Quantity}\;=\;10^{\left(CT-b\right)/m}$$

The ratios of the quantity of candidate gene/average quantity of housekeeping genes were subsequently calculated for each candidate gene and used to assess the differences in expression levels between experimental groups.

#### 2.4.4. Anti-Inflammatory Effect of EE

To evaluate the anti-inflammatory properties of EE, a similar experiment was designed using 10 µM CuSO_4_ to stimulate inflammation. After 4 and 24 h of CuSO_4_ exposure, the expression of genes related to inflammatory processes were analyzed in zebrafish larvae using qPCR.

The resulting cDNA was used to measure the expression of 9 immune system genes in a real-time quantitative polymerase chain reaction (qPCR). Two housekeeping genes (*ß-actin* and *efl1-α*) were used as the reference. The expression of genes encoding pro-inflammatory cytokines (*il-1ß*, *il-8*, and *tnf-α*), neutrophil (*mpo*), complement 3a (*c3a*), phospholipase A2 (*pla2*), *cox-2*, transcription factor NF-κB (*nf-ƙb*), and an anti-inflammatory response cytokine (*il-10*) were evaluated. The list of specific primers used is given in Table 1. Primers were designed on Primer3 software. Amplifix software was used to check the quality of the primers. The efficiency of each primer was confirmed by RT-PCR.

### 2.5. Data Presentation and Statistical Analyses

Data analyses were performed using SPSS 16.0 (SPSS Inc, Chicago, Illinois, United States). In the case of data with a normal distribution, data are shown as mean ± SEM; a *t*-test and one-way analysis of variance with LSD post hoc test were used for statistical comparisons to determine treatment differences. A probability level of *p* < 0.05 was considered significant. 

When data were not normally distributed, the non-parametric Kruskal–Wallis one-way analysis of variance by ranks was performed, followed by a Mann–Whitney test to determine significant differences between the experimental groups. When non-parametric statistics were used, the data are presented using Turkey’s boxplot (bottom and top of the box are 25th and 75th percentile, the horizontal line in the box is the median, the ends of the whiskers represent 1.5 times the interquartile distance, and the individual dots are values that fall outside the whiskers).

## 3. Results

### 3.1. Protective Activity of EE against CuSO_4_ Toxicity in Zebrafish Larvae

**Figure 1:**
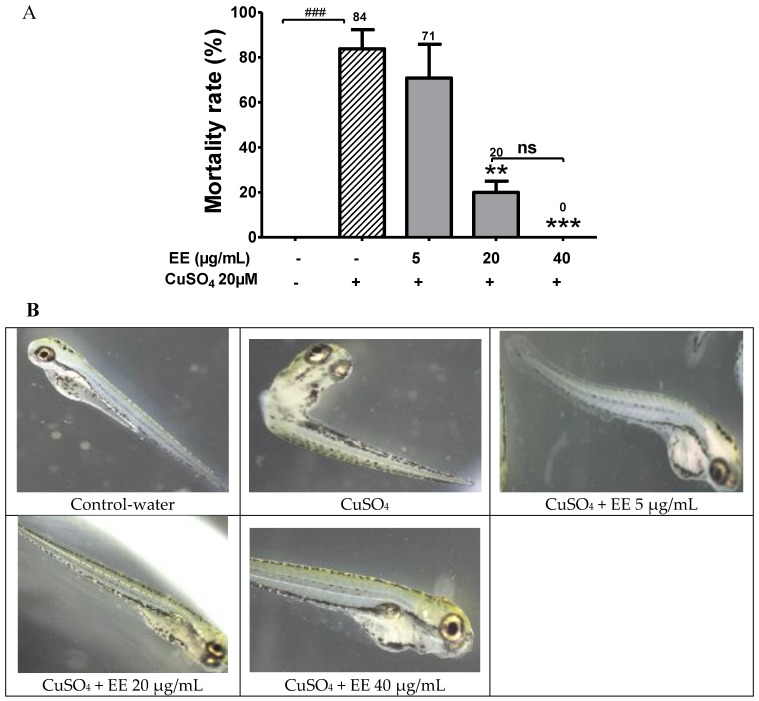
Protective effect of the ethanol extract of *C. cyrtophyllum* leaves (EE) against CuSO_4_ toxicity. (**A**) Mortality rates of zebrafish larvae after treatment with 20 µM CuSO_4_ and ethanol extracts of C. cyrtophyllum (EE) at different doses. (**B**) Representative photographs of zebrafish larvae treated with 20 µM CuSO_4_ and ethanol extracts of *C. cyrtophyllum* (EE) at different doses. The data are presented as mean ± S.E. for three different experiments performed in triplicate. ### *p* < 0.001, ** *p* < 0.01, and *** *p* < 0.001 compared to the CuSO_4_ alone group.

In the present study, we showed that the exposure of CuSO_4_ at high dose of 20 µM induced mortality in zebrafish larvae (Figure 1A), and EE at the doses of 20 and 40 µg/mL effectively inhibited the toxicity of CuSO_4_, improving fish survival. The mortality of larvae which were treated with CuSO_4_ alone was 84%, while the mortality rate was 20% for co-administration with EE at the 20 µg/mL dose. At the high EE dose 40 µg/mL, no dead larvae were observed after 24 h. 

The protective effect of EE against CuSO_4_ was further confirmed by representative photographs of zebrafish larvae treated with 20 µM CuSO_4_ and EE at different doses. Photographs of zebrafish showed that treatment only with CuSO_4_ induced toxicity in larvae. Several malformations were observed, including trunk curvature, shorter body length, and loss of moving ability. Interestingly, in the groups that were treated with copper plus EE, the extent of morphological abnormality decreased. At high dose, EE 40 µg/mL protected zebrafish larvae against toxicity of CuSO_4,_ and no morphological abnormality was observed (Figure 1B). These results provide evidence that EE protected larvae from CuSO_4_ toxicity in a dose-dependent manner. To elucidate the protective mechanisms of EE, we further evaluated its antioxidant and anti-inflammatory activities.

### 3.2. Antioxidant Effect

#### 3.2.1. In Vitro Antioxidant Activity of EE

##### Effect of the Ethanol Extract of C. cyrtophyllum Leaves (EE) on DPPH Radical Scavenging 

The free-radical scavenging ability of EE is shown in Table 2. The positive control, quercetin, has a strong DPPH scavenging property with a maximum achievable response (Emax) of 93.82%. The concentration that gave the half-maximal effect (IC_50_) was 3.93 ± 1.09 µg/mL. Similar to quercetin, EE showed a strong antioxidant activity with an IC_50_ value of 16.45 ± 1.11 μg/ mL and an Emax of 94.24%. The results indicate that EE may act as a DPPH radical scavenger.

##### Effect of the Ethanol Extract of C. cyrtophyllum Leaves (EE) on Cu^2+^ Chelation Ability

**Figure 2:**
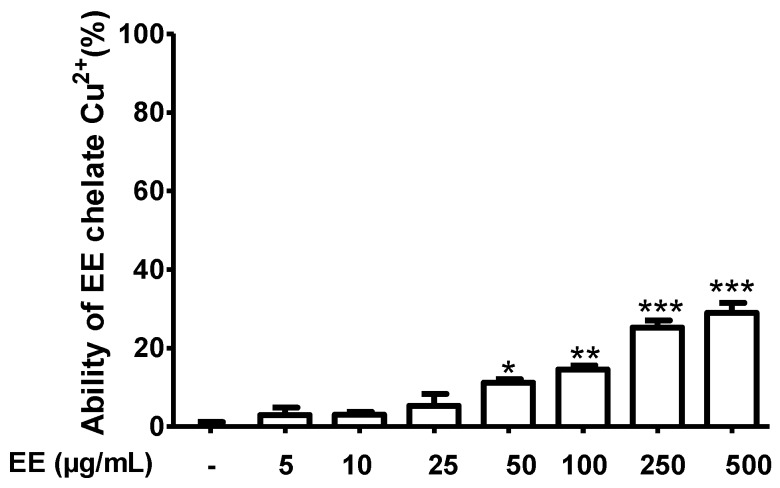
Effect of the ethanol extract of C. cyrtophyllum leaves (EE) on Cu^2+^ chelation ability. Each bar represents the mean ± S.E. for three different experiments performed in triplicate. * *p* < 0.05, ** and *** *p* < 0.001 compared to the control group.

The ability of EE to chelate Cu^2+^ is shown in Figure 2. Compounds in EE bind Cu^2+^ with the formation of a complex EE–Cu^2+^. EE showed a weak copper chelating activity. At the highest concentration of 500 µg/mL, Cu^2+^ chelating ability of EE only reached 29%. 

#### 3.2.2. Effect of the Ethanol Extract of *C. cyrtophyllum* Leaves (EE) on the Generation of Reactive Oxygen Species

**Figure 3:**
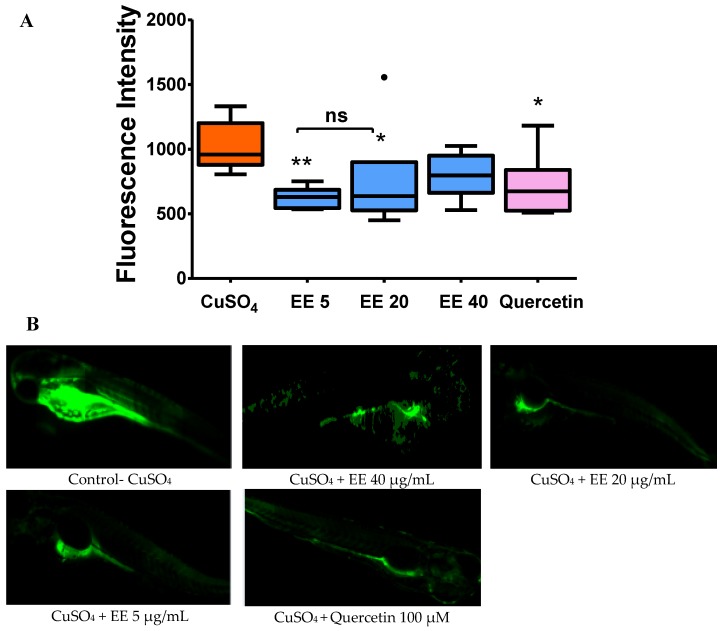
Inhibitory effect of ethanol extract (EE) from leaves of *C. cyrtophyllum* Turcz on CuSO_4_-stimulated ROS production in zebrafish larvae. The zebrafish were exposed to different concentrations of EE (5, 20, and 40 µg/mL) or 100 µM quercetin for 1 h and then exposed to 10 µM CuSO_4_ for 20 min to induce oxidative stress. After 20 min, larvae were incubated with H2DCFDA (20 µg/mL) for 1 h. Fluorescent probes were used to detect and quantify oxidative stress by measuring the fluorescent intensity (FI) in larvae using Autovision software and a BD pathway 855 system (BD Biosciences). (**A**) Fluorescence intensity obtained from individual zebrafish larvae. The data are presented as medians for six different larvae, * *p* < 0.05 and ** *p* < 0.01 compared to the CuSO_4_ alone Group. (**B**) Representative fluorescence micrographs of ROS production.

As shown in Figure 3, quercetin 100 µM inhibited ROS formation, thus fluorescence intensity significantly decreased compared to the CuSO_4_ group. Similar to quercetin, co-administration with EE at 5 and 20 µg/mL significantly decreased fluorescence intensity. These results suggest that administration of EE can limit the generation of reactive oxygen species generated in response to CuSO_4_.

#### 3.2.3. Effect of the Ethanol Extract of *C. cyrtophyllum* Leaves (EE) on Antioxidant Gene Expression

**Figure 4:**
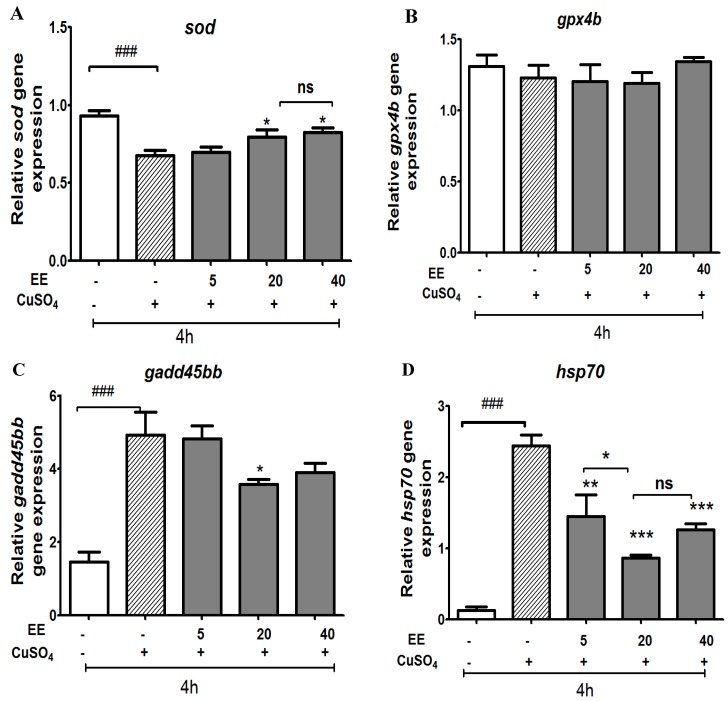
Effect of ethanol extract (EE) of *C. cyrtophyllum* Turcz leaves on expression of antioxidant genes: *sod* (**A**); *gpx4* (**B**); hsp70 (**C**), and *gadd45bb* (**D**). The zebrafish larvae were exposed to EE for 1 h and to CuSO_4_ 10 µM for 4 h. After 4 h, larvae were collected for qPCR analysis. The relative gene expressions are presented as the ratio of the quantity of candidate gene/average quantity of housekeeping genes. A pool of 20 larvae per group (n = 3) was used. Each bar represents the mean ± S.E. for three different experiments performed in triplicate. * *p* < 0.05, ** and ### *p* < 0.01, and *** *p* < 0.001 compared to the CuSO_4_ alone group.

To further investigate the mechanism of antioxidant action, we measured the effects of EE on the expression of two genes involved in antioxidant systems (*sod* and *gpx4*). As shown in Figure 4, exposure to copper sulfate resulted in a significant reduction in *sod* compared to the control group. EE at 20 and 40 µg/mL significantly increased *sod*, whereas expression of *gp4x* was not affected by any treatment.

We also designed experiments to examine whether copper sulfate is capable of inducing stress response or growth arrest and DNA damage through evaluating the expression levels of a growth arrest and DNA-damage gene (*gadd45bb*) and a stress-related gene (*hsp70)*. As shown in Figure 4, CuSO_4_ induced a stress response; the mRNA expression levels of *hsp70* and *gadd45bb* were significantly upregulated. The co-administration of EE at the doses of 5, 20, and 40 µg/mL protected zebrafish larvae against oxidative damage due to CuSO_4_ and therefore the expressions of *hsp70* and *gadd45bb* were down-regulated.

### 3.3. Anti-Inflammatory Effect of EE

#### 3.3.1. Effect of the Ethanol Extract of *C. cyrtophyllum* Turcz Leaves on the Expressions of *c3a*, *cox-2*, and *pla2* Genes

**Figure 5:**
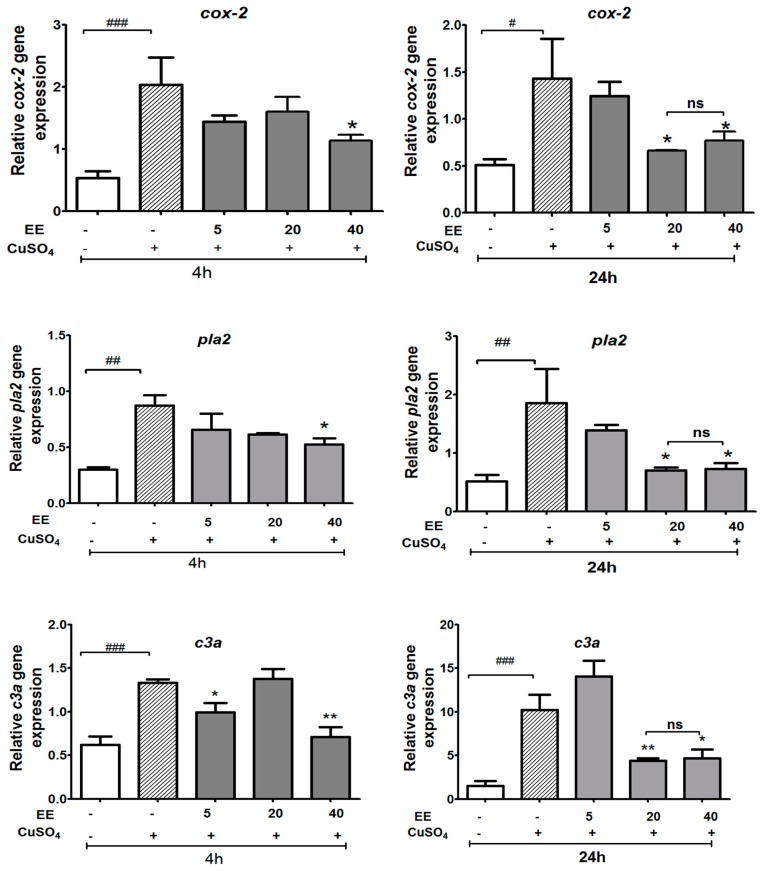
Effect of ethanol extract (EE) of *C. cyrtophyllum* Turcz leaves on the expression of genes in the eicosanoid pathway (*pla2*, *cox-2*, and *c3a.*). The zebrafish larvae were exposed to EE for 1 h and CuSO_4_ 10 µM for 4 and 24 h. After 4 or 24 h, larvae were collected for qPCR analysis. The relative gene expressions are presented as the ratio of the quantity of candidate gene/average quantity of housekeeping genes. A pool of 20 larvae per group (n = 3) was used. Each bar represents the mean ± S.E. for three different experiments performed in triplicate. * *p* < 0.05, ** and ### *p* < 0.01, and *** *p* < 0.001 compared to the CuSO_4_ alone group.

For the expression of two genes involved in the eicosanoid pathway (*cox-2* and *pla2*), and of the complement gene *c3a*, as shown in Figure 5, CuSO_4_ markedly increased the expression of *cox-2*, *c3a*, and *pla2* genes when compared with the control group. EE at 40 µg/mL greatly suppressed the CuSO_4_-induced mRNA levels of *cox-2*, *pla2*, and *c3a* at both 4 and 24 h after copper sulfate exposure, while EE at 20 µg/mL only showed an effect on expression after 24 h.

#### 3.3.2. The EE Inhibited the Transcription of CuSO_4_-Induced Pro-Inflammatory Cytokines in Zebrafish Larvae

**Figure 6:**
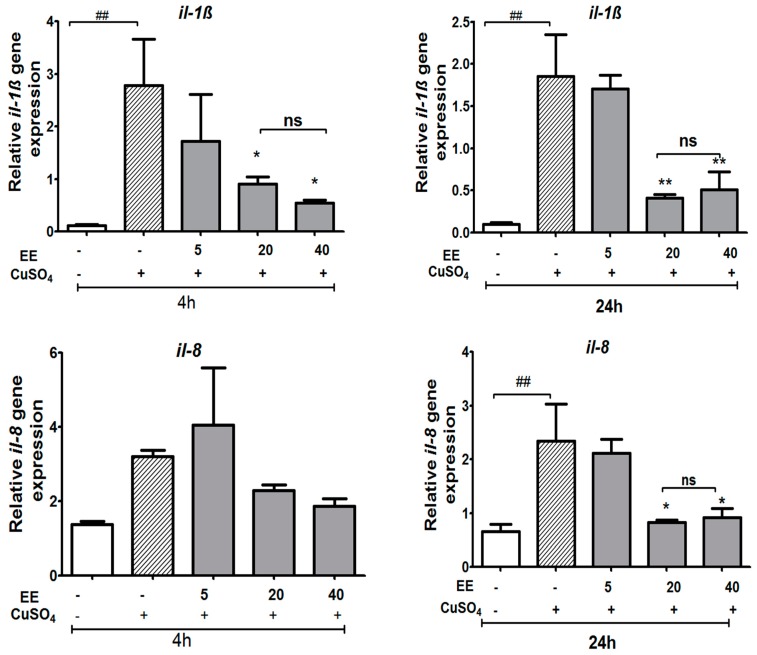
Relative expression of genes involved in immune responses (*il-1ß* and *il-8*) of zebrafish larvae after exposure to CuSO_4_ and treatment with the ethanol extract (EE) of *C. cyrtophyllum* Turcz at different doses. The zebrafish larvae were exposed to EE for 1 h and to CuSO_4_ 10 µM for 4 and 24 h. After 4 or 24 h, larvae were collected for qPCR analysis. The relative gene expressions are presented as the ratio of the quantity of candidate gene/average quantity of housekeeping genes. A pool of 20 larvae per group (n = 3) was used. Each bar represents the mean ± S.E. for three different experiments performed in triplicate. * *p* < 0.05, ** and ### *p* < 0.01, *** *p* < 0.001 compared to the CuSO_4_ alone group.

**Figure 7:**
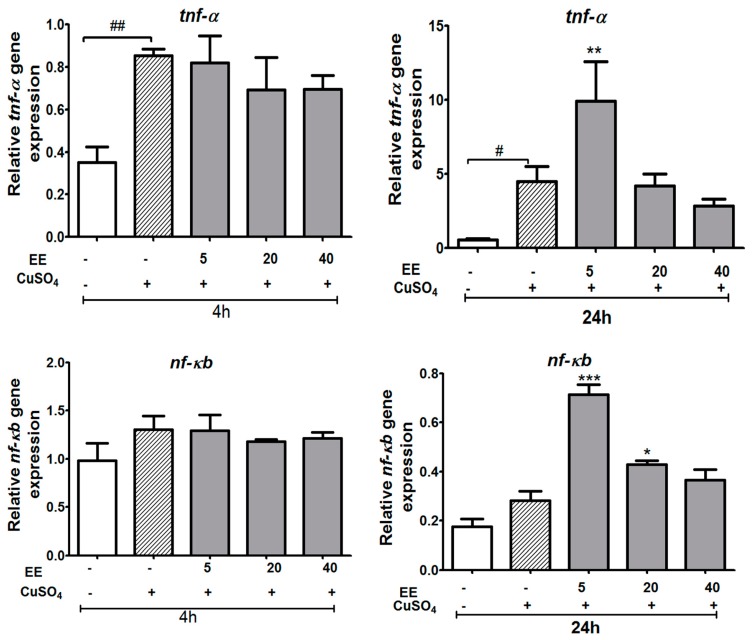
Relative expression of genes involved in immune responses (*tnf-α* and *nf-ƙb*) of zebrafish larvae after exposure to CuSO_4_ and treatment with the ethanol extract (EE) of *C. cyrtophyllum* Turcz at different doses. The zebrafish larvae were exposed to EE for 1 h and to CuSO_4_ 10 µM for 4 and 24 h. After 4 or 24 h, larvae were collected for qPCR analysis. The relative gene expressions are presented as the ratio of the quantity of candidate gene/average quantity of housekeeping genes. A pool of 20 larvae per group (n = 3) was used. Each bar represents the mean ± S.E. for three different experiments performed in triplicate. * *p* < 0.05, ** and ### *p* < 0.01, and *** *p* < 0.001 compared to the CuSO_4_ alone group.

We examined whether the expression of pro-inflammatory cytokine genes increased due to CuSO_4_ exposure and, subsequently, whether EE inhibits CuSO_4_-induced expression of *tnf-α*, *il-1β*, *nf-ƙb*, and *il-8* at concentrations of 5, 20, and 40 µg/mL. Real-time RT-PCR analysis revealed that CuSO_4_ stimulated the inflammation process by a significant increase of *il-1β* and *tnf-α* expression at 4 and 24 h and *il-8* levels at 24 h after copper sulfate exposure (Figure 6 and Figure 7). The co-administration of EE at the doses of 20 and 40 µg/mL resulted in a decrease in the mRNA levels of *il-1β* at both 4 and 24 h, and *il-8* at 24 h (Figure 6). No significant differences were observed for the expression of *nf*-*ƙb* or *tnf-α* between the different treatment groups (Figure 7). These results suggest that the EE at concentrations of 20 and 40 µg/mL inhibited transcription activities of pro-inflammatory cytokines induced by CuSO_4_ in zebrafish larvae.

#### 3.3.3. Effect of the Ethanol Extract (EE) of *C. cyrtophyllum* Turcz Leaves on Expression of the *mpo* Gene

**Figure 8:**
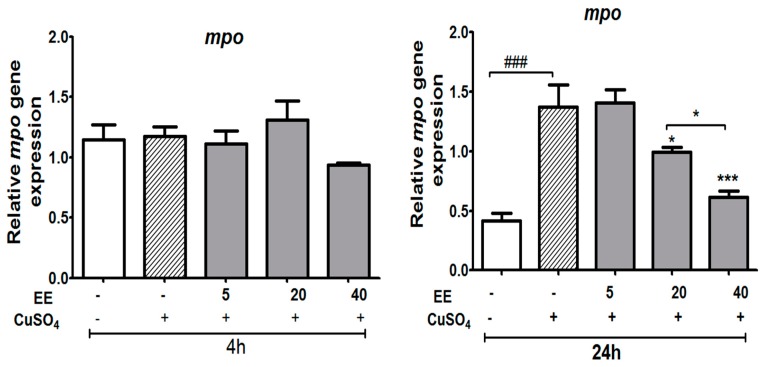
Relative expression of genes involved in immune responses (*mpo*) of zebrafish larvae after exposure to CuSO_4_ and treatment with the ethanol extract (EE) of *C. cyrtophyllum* Turcz. The data are presented as mean ± S.E. for three different experiments performed in triplicate. * *p* < 0.05, ** and ### *p* < 0.01, *** *p* < 0.001 compared to the CuSO_4_ alone group.

Neutrophil migration, as indirectly measured by the mRNA level of *mpo*, displayed a significant increase after 24 h of exposure to copper sulfate. Exposing zebrafish larvae to EE resulted in a decrease in the mRNA levels of *mpo* at 20 and 40 µg/mL (*p* < 0.05) (Figure 8).

#### 3.3.4. Effect of the Ethanol Extract (EE) of *C. cyrtophyllum* Turcz leaves on the Expression of Anti-Inflammatory Cytokine *il-10*

**Figure 9:**
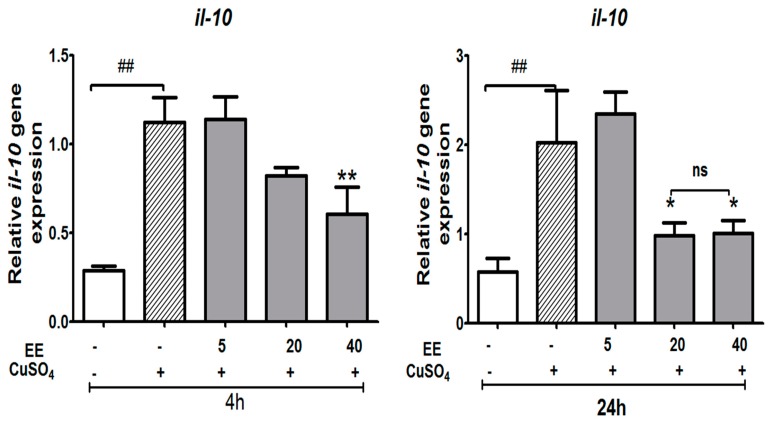
Relative expression of anti-inflammatory gene (*il-10)* of zebrafish larvae after exposure to CuSO_4_ and treatment with the ethanol extract (EE) of *C. cyrtophyllum* Turcz at different doses. The zebrafish larvae were exposed to EE for 1 h and to CuSO_4_ 10 µM for 4 and 24 h. After 4 or 24 h, larvae were collected for qPCR analysis. The relative gene expressions are presented as the ratio of the quantity of candidate gene/average quantity of housekeeping genes. A pool of 20 larvae per group (n = 3) was used. Each bar represents the mean ± S.E. for three different experiments performed in triplicate. * *p* < 0.05, ** and ### *p* < 0.01, and *** *p* < 0.001 compared to the CuSO_4_ alone group.

As shown in Figure 9, stimulating zebrafish larvae with CuSO_4_ increased the level of *il-10* mRNA. Increased *il-10* expression in response to CuSO_4_ stimulation alone showed the regulatory action on cells after the inflammatory reaction. The level of *il-10* was significantly suppressed by EE at 40 µg/mL at 4 h, and at both 20 and 40 µg/mL at 24 h (*p* < 0.05). The level of *il-10* decreased the most under the EE treatment condition, which illustrated the effect of EE on reinforcing cellular immunity and inhibiting humoral immunity.

## 4. Discussion

### 4.1. Protective Activity of the Ethanol Extract of *C. cyrtophyllum* against CuSO_4_ Toxicity

Copper is an essential micronutrient but misregulation of intracellular levels can become toxic to many cell types. Copper generates dose-dependent responses. A previous study demonstrated that low concentrations of copper (<10 µM) did not induce mortality [16]. At the concentration of 10 μM, although it did not cause a significant decrease of zebrafish larvae survival, copper sulfate was able to induce oxidative stress and a marked increase of inflammatory markers [17]. A concentration of 25 μM induced a 75–80% death rate [18]. In the present study, CuSO_4_ at 20 µM induced 80% mortality after 24 h of exposure. Interestingly, treatment with EE at concentrations of 20 and 40 µg/mL showed a protective effect against damage induced by CuSO_4_.

What is the reason for this? Which molecular mechanism of EE helped zebrafish larvae to avoid the toxicity of copper? To better understand the protective mechanism of EE, the toxicity mechanism of copper in zebrafish larvae first needs to be identified.

It was suggested recently that copper induces oxidative stress [16,18]. Oxidative stress can occur by two mechanisms: the direct effect of copper, which induces DNA and cell damage, or the increase of ROS release due to the activation of phagocytic cells and increased tissue damage [13]. The increase of ROS release might induce the activation of activator protein-1 (AP-1) and nuclear factor kappa-B (Nf-κB), both signaling pathways that upregulate pro-inflammatory cytokines and chemokines leading to inflammation. Inflammation represents the result of copper damage. Both oxidative stress and inflammation are related to the toxicity mechanism of copper. We thus investigated the antioxidant and anti-inflammatory properties of EE.

### 4.2. Antioxidant and Anti-Inflammatory Activity of the Ethanol Extract of C. cyrtophyllum

To evaluate the antioxidant activity, we first used an in vitro test, the DPPH assay. DPPH radical scavenging assay is one of the most common methods used to evaluate the radical scavenging activity of antioxidants because of its speed, reliability, and reproducibility. A change in color from purple to yellow indicates a decrease in absorbance of DPPH radicals. This demonstrates that an antioxidant present in a solution is interacting with the free radicals [11]. In this study, EE exhibited strong antioxidant properties against 1,1-diphenyl,2-picryl hydrazyl (DPPH), with an IC_50_ value of 16.45 ± 1.11 µg/mL. The antioxidant activity of EE found here was stronger than that reported by Liu et al. (2011) for a methanol extract of *C. cyrtophyllum* (52.74 ± 2.07 µg/mL) [9]. This can be explained by the fact that flavonoids are important antioxidants due to their high redox potential, which allows them to act as reducing agents, hydrogen donors, and singlet oxygen quenchers [19]. Antioxidant activity was correlated with total flavonoids. The higher is the content of flavonoids, the stronger is the antioxidant capacity in vitro. In our study, EE was found to present higher flavonoid contents than the methanol extract reported by Liu et al. [9]. Another antioxidant mechanism of flavonoids may result from the interactions between flavonoid and metal ions (especially iron and copper) leading to chelates formation that are only slightly active in the promotion of free-radical reaction. In our study, EE can chelate copper, decreasing the toxicity of CuSO_4_. However, the copper chelating ability of EE is weak. It did not appear as a major mechanism for protective activity of the ethanol extract of *C. cyrtophyllum* against CuSO_4_ toxicity.

Although the in vitro model is simple, fast, and inexpensive, it cannot be a complete substitute for in vivo animal testing. The absence of biokinetics in in vitro methods may lead to a misinterpretation of the data. Animal models thus are more reliable than in vitro tests [20]. Among animal models, zebrafish display several pathological features similar to those of humans [21]. In this study, we used a zebrafish model to confirm the antioxidant effects of EE, as well as to elucidate the molecular mechanisms of its antioxidant activity.

There are two main mechanisms of drug-induced oxidative stress: an increase in ROS production and a reduction of cellular antioxidant genes. We thus examined the effects of EE on ROS production as well as on the expression of antioxidant genes.

Regarding ROS production, the image analysis in stress-induced ROS generation showed that copper sulfate induced toxicity in the cells via the generation of reactive oxygen species (ROS). EE displayed a protective effect against stress in vivo by reducing ROS formation in zebrafish larvae. Regarding the expression of antioxidant genes, in our study, *sod* was decreased significantly after 4 h of exposure to copper when compared to control, while groups that were treated with CuSO_4_ plus EE at 20 and 40 µg/mL resulted in a significant increase of mRNA levels of *sod* when compared with the CuSO_4_ group. Increasing superoxide dismutase (SOD) helps to transfer ROS to hydrogen peroxide (H_2_O_2_), which then forms a non-toxic compound (H_2_O) under the effects of catalase, glutathione peroxidase, and peroxidase.

Major cytotoxic roles for ROS include the activation of the apoptosis pathway [22]. Heat shock proteins are one of the key players in protecting the cell against the harmful effects of oxidative stress in the cellular stress response process and are crucial for defending cells from copper toxicity [16]. Our results are similar to those previously reported where copper induces *hsp70* expression [16]. Interestingly, expression of *hsp70* decreased in the EE treatment groups. This result suggests that the presence of EE helped to protect zebrafish larvae against CuSO_4_ toxicity, reducing oxidative stress and damage. As a result, the expression of *hsp70* decreased.

Gadd45 participates in cell growth and cell cycle control, DNA repair, apoptosis, maintenance of genomic stability, and the regulation of signaling pathways. Transcription of the *gadd45* genes is induced by DNA-damaging agents and other cellular stresses and is associated with growth arrest [23,24]. Olivari et al. (2008) described events induced by copper via oxidative stress, such as cell death by apoptosis and necrosis in hair cells of the lateral line of zebrafish [13]. Supporting these data, our results show that copper induced activation of the apoptosis pathway in such a way that mRNA expression level of *gadd45bb* was significantly upregulated. EE can scavenge superoxide anions, inhibit ROS production, and defend cells from copper toxicity, decreasing cellular stresses, apoptosis, and DNA-damage. As a result, the expression of *gadd45bb* was downregulated compared to the CuSO_4_ alone group.

Intracellular production of reactive oxygen species (ROS) is deeply involved in inflammatory responses. High levels of redox metals promote ROS formation and these metals can also act as mediators of inflammation, inducing peripheral inflammation in numerous models [25]. Leite et al. (2013) reported the gradual accumulation of copper in zebrafish larvae within 24 hpf; increased activity of NO; upregulation of pro-inflammatory cytokine-related genes *il-1β*, *tnf*, and *cox-2*; and increased PGE2 and myeloperoxidase (MPO) levels, which are also known for their activity as inflammatory players [18]. Similar to previous studies, our data also show that copper induced inflammation through the upregulation of *cox-2*, *il-1β*, *tnf-α*, *mpo*, *pla2*, *il-8*, *c3a*, and *il-10.* Altogether, this study provides more evidence on the effects of copper as well as on the general scenario of the inflammatory status and response to copper exposure that corroborates copper’s role in oxidative stress and inflammation.

The expression of *cox-2, pla2, c3a, il-1ß*, and *il-8* decreased in the EE treatment groups. COX-2 and PLA2 are involved in the synthesis of key biological mediators in inflammation. Inhibition of these enzymes is a well-established target in the discovery of anti-inflammatory drugs, with COX-inhibitors being the most prominent drug class in non-steroidal anti-inflammatory drugs (NSAIDs). Flavonoids play an important role in various medicinal applications. C. *cyrtophyllum* leaves present high flavonoid contents [8,9]. Flavonoids have been reported to inhibit the activities of arachidonic acid metabolizing enzymes such as PLA2, COX, and 5-LOX, to reduce the production of inflammatory metabolites from arachidonic acid and oxidative damage [26]. IL-1ß and IL-8 are pro-inflammatory cytokines. There is abundant evidence that certain pro-inflammatory cytokines such as IL-1β, IL-2, TNF-α, IL-6, IL-8, and IFN-γ are involved in the process of pathological inflammation. Because of this, the cytokine system constitutes a very interesting target for the development of clinically relevant anti-inflammatory drugs. In this study, EE effectively inhibited the production of *il-1ß* and *il-8* at both 20 and 40 µg/mL by suppressing their mRNA expression in CuSO_4_-stimulated zebrafish larvae. This result demonstrates that EE effectively inhibits the generation of an inflammatory response. However, the level of TNF-α transcription was not affected by EE at 4 h. At 24 h, expression of *tnf-α* even increased at the dose of 5 µg/mL. Increased expression of *tnf-α* can explain the weak anti-inflammatory activity of EE at 5 µg/mL.

The regulation of inflammatory gene transcription is controlled by specific signaling pathways and transcription factors, such as NF-κB and AP-1. In response to stimulation, NF-κB is liberated from a complex with I-κB and induces transcription of inflammatory genes that cause acute inflammation and a systemic inflammatory response syndrome [27]. In this study, CuSO_4_ did not increase the expression of *nf-κb*. This result demonstrates that *nf-κb* did not regulate the inflammatory gene transcription stimulated by CuSO_4_. The increase expression of inflammatory genes due to CuSO_4_ can relate to other transcription factors with other signaling pathways that need further studies. The ethanol extract from *C. cyrtophyllum* had no effect on the expression of *nf-κb* at 4 h; however; expression of *nf-κb* increased at the dose of 5 and 20 µg/mL at 24 h. In our previous study, EE alone had no effect on the expression of *nf-κb*. Increasing its expression when association with CuSO_4_ may be related to another function of *nf-κb*. The association of NF-kB activity and inflammatory disease is not easy to interpret because both pro- and anti-inflammatory mediators are produced during inflammation [28]. In 2001, Lawrence et al. [29]. showed NF-kB involves in both the onset and resolution of acute inflammation in a single model system using pharmacological inhibitors. These studies confirmed the role of NF-kB in proinflammatory gene induction but also showed a role for NF-kB in the expression of anti-inflammatory genes during the resolution of inflammation [29]. Thus, increasing expression of *nf-κb* at the dose of 5 and 20 µg/mL at 24 h may be related to resolution function of NF-kB in a cute inflammation.

However, the increase in mRNA levels is not directly proportional to the amount of protein translated, due to transcriptional and post translational modifications. Although no significant differences in the mRNA levels of *tnf-α* and *nf-κb* at 4 h or increase of the expression of these genes at 24 h was observed, further studies are required in order to investigate the expression at the protein level and to verify the presence and activation of these proteins.

Recently, d’Alencon et al. demonstrated that copper sulfate exposure induces neutrophil migration to the inflammatory focus, in response to damage induced in hair cells of the lateral line of zebrafish larvae [30]. Activated neutrophils, monocytes, and some tissue macrophages release MPO at the site of inflammation. MPO catalyzes the reaction between hydrogen peroxide (H_2_O_2_) and physiological halides (such as Cl^−^) to form reactive oxidants such as hypochlorous acid (HOCl) [31]. In this study, EE effectively inhibited the expression of *mpo* at 20 and 40 µg/mL at 24 h. This suggests that EE has an anti-inflammatory role through the inhibition of leukocyte infiltration.

Interleukin-10 (IL-10) is a cytokine with potent anti-inflammatory properties that plays a central role in limiting the host immune response to pathogens, thereby preventing damage to the host and maintaining normal tissue homeostasis. IL-10 is a molecule that displays both immunostimulatory and immunoregulatory activities [32]. IL-10 can up-regulate endogenous anti-cytokines and down-regulate pro-inflammatory cytokine receptors to dampen uncontrolled production of inflammatory cytokines and excessive inflammation during infection [33]. In this study, stimulation of zebrafish larvae with CuSO_4_ increased the level of *il-10* mRNA at 4 and 24 h after exposure. The elevation of *il-10* displayed immunoregulatory activity on cells after the inflammatory reaction [18]. Co-administration with EE at 20 and 40 µg/mL down-regulated *il-10.* Supporting these data, a previous study conducted by Visser et al. (1998) revealed that elevated *il-10* levels were also observed in an LPS-induced inflammatory model in peritoneal macrophages, demonstrating similarities of modulation mechanisms in these two models [34].

### 4.3. The Relation between Dose and Response

In most cases, EE displayed an effect on gene expression depending on the dose. At the low dose of 5 µg/mL, EE generally exerted no detectable effects. However, at 20 µg/mL, EE showed a higher effect. Interestingly, the anti-inflammatory property of EE not only depends on the dose but also on the time. At 4 h, EE at 20 µg/mL indicated no effects or low effect on the inhibition expression of inflammatory genes. However, this effect increased at 24 h. One possible explanation could be that, as exposure to the extract is prolonged over time, there is an increase in the accumulation of the extract until it reaches a concentration that induces anti-inflammatory effects in zebrafish. At the highest dose of 40 µg/mL, EE induced the best effects at both 4 and 24 h.

### 4.4. The Potential Area of Application of EE

The results of this study suggest that the ethanol extract of *C. cyrtophyllum* leaves possesses antioxidant and anti-inflammatory activities. They provide some evidence for the use of the plant extract in the prevention as well as treatment of oxidative stress and inflammatory conditions. However, before applying EE in treatment human diseases, we still need to do experiments in other models to confirm the anti-inflammatory and antioxidant activities of EE. We also need to perform additional experiments to determine whether EE is safe for testing in human subjects; preclinical toxicology studies need to be performed to identify the treatment regimen associated with the least degree of toxicity and thus determine a suitable and safe starting dose for clinical trials.

## 5. Conclusions

The ethanol extract of *C. cyrtophyllum* displayed prominent antioxidant effects through radical scavenging activity in vitro and decreased production of ROS in a CuSO_4_-induced zebrafish inflammation model. In addition, in vivo results in zebrafish suggest that the ethanol extract from *C. cyrtophyllum* also inhibits oxidative stress via the upregulation of *sod* and the downregulation of *hsp70* and *gadd45bb*. EE also inhibited inflammation via the downregulation of the inflammatory genes *cox-2*, *pla2*, *c3a*, *il-1*, *il-8*, *mpo*, and *il-10*. These in vivo results help to elucidate the protective mechanism against CuSO_4_ toxicity of the ethanol extract of leaves of *C. cyrtophyllum*. Taken together, these findings provide a pharmacological basis for the use of *C. cyrtophyllum* leaves in the treatment of inflammatory disorders.

## Figures and Tables

**Table 1:** Primer pairs used in this study.

Gene Name	Function	GenBank Accession No.	Forward and Reverse Primer Sequences (5′-3′)
β-actin	Housekeeping gene	AF057040	*Fwd:* CCCCATTGAGCACGGTATTG *Rev*: ATACATGGCAGGGGTGTTGA
Elongation factor 1 alpha (*efl1-α*)	Housekeeping gene	L23807.1	*Fwd:* CCAAGGAAGTCAGCGCATAC *Rev*: CCTCCTTGCGCTCAATCTTC
Interleukin-1 *(il-1ß*)	Pro-inflammatory cytokine	NM_212844.2	*Fwd:* AAAGTGCGCTTCAGCATGTC *Rev:* GCTGGTCGTATCCGTTTGGA
Interleukin -8 *il-8* (*cxcl8b.1*)	cytokine	NM_001327985.1	*Fwd:* GCCTTCATGCTTCTGATCTGC *Rev*: AATCACCCACGTCTCGGTAGGA
Cyclooxygenase -2 (*ptgs2a* or *cox2*)	Catalyze the formation of prostaglandin, thromboxane	NM_153657.1	*Fwd:* ACAGATGCGCTACCAGTCTT *Rev*: CCCATGAGGCCTTTGAGAGA
Phospholipase A2 *pla2* (*pla2g4aa*)	Provide precursors for generation of eicosanoids	NM_131295.2	*Fwd:* TCATGTCTCCTGGGCTGTTT *Rev*: CCAGCTCCTCCTCCATAGTG
Tumor necrosis factor *(tnf-α)*	Pro-inflammatory cytokine	AB183467	*Fwd:* CACAAAGGCTGCCATTCACT *Rev*: GATTGATGGTGTGGCTCAGGT
*nf-* *ƙb (nkap)*	A ubiquitous transcription factor	NM_001003414.1	*Fwd:* GGTCGGACAGAGATCACGGATT *Rev*: TGCTGTTCTTCACGTCCTCT
Interleukin -10 (*il-10*)	Anti-inflammatory cytokine	AY887900.1	*Fwd:* AGTCATCCTTTCTGCTCTGCT *Rev*: AAAGCCCTCCACAAATGAGC
*c3a (c3a.1)*	Complement 3a	NM_131242.1	*Fwd: GTACGAGGCGAACAACTGGA* *Rev: CATCATACGCCGCAGCTTTC*
*mpo*	Myeloperoxidase	AF349034.1	*Fwd*: GTGGTCGTGTCGGTTCTCTT *Rev*: GCAGATTATGCGGGCCATTG
*sod (sod1)*	Superoxide dismutases	NM_131294.1	*Fwd*: ATGGTGAACAAGGCCGTTTG *Rev*: AAAGCATGGACGTGGAAACC
*gpx4b*	glutathione peroxidase	BC095133.1	*Fwd:* TGAGAAGGGTTTACGCATCCTG *Rev*: TGTTGTTCCCCAGTGTTCCT
*hsp70*	Protect cell from oxidative stress	AF210640.1	*Fwd:* CAACGTGCTGATCTTTGACC *Rev*: TCCTCTTGGCTCGTTCACAT
*gadd45bb*	Growth arrest and DNA-damage-inducible	NM_001012386.2	*Fwd:* CGCTTCAGATCCACTTCACG *Rev*: TCCCACTTCCTTCAGCTTGA

**Table 2:** DPPH radical scavenging activity of EE.

	EE	Quercetin
**Emax (%)**	94.24	93.82
**IC_50_ (µg/mL**)	16.45 ± 1.11	3.93 ± 1.09
**95% Confidence Interval**	11.63–23.27	2.98–5.17
**R^2^**	0.9958	0.98995
**Number of Points Analyzed**	7	7
